# Lessons from a breast cell annotation competition series for school pupils

**DOI:** 10.1038/s41598-022-11782-9

**Published:** 2022-05-12

**Authors:** Wenqi Lu, Islam M. Miligy, Fayyaz Minhas, Young Saeng Park, David R. J. Snead, Emad A. Rakha, Clare Verrill, Nasir Rajpoot

**Affiliations:** 1 Department of Computer Science, University of Warwick, Coventry, UK; 2 Nottingham Breast Cancer Research Centre, Division of Cancer and Stem Cells, School of Medicine, Nottingham City Hospital, University of Nottingham, Nottingham, UK; 3 Department of Pathology, Faculty of Medicine, Menoufia University, Shibin El Kom, Egypt; 4 Warwick Manufacturing Group (WMG), University of Warwick, Coventry, UK; 5 Cellular Pathology, University Hospitals Coventry and Warwickshire NHS Trust, Coventry, UK; 6 Department of Cellular Pathology, Oxford University Hospitals NHS Foundation Trust, Oxford, Oxfordshire UK; 7 NIHR Oxford Biomedical Research Centre, Oxford University Hospitals NHS Foundation Trust, Oxford, Oxfordshire UK; 8 Nuffield Department of Surgical Sciences, Oxford University, Oxford, Oxfordshire UK; 9 The Alan Turing Institute, London, UK

**Keywords:** Medical research, Data acquisition

## Abstract

Due to COVID-19 outbreaks, most school pupils have had to be home-schooled for long periods of time. Two editions of a web-based competition “Beat the Pathologists” for school age participants in the UK ran to fill up pupils’ spare time after home-schooling and evaluate their ability on contributing to AI annotation. The two editions asked the participants to annotate different types of cells on Ki67 stained breast cancer images. The Main competition was at four levels with different level of complexity. We obtained annotations of four kinds of cells entered by school pupils and ground truth from expert pathologists. In this paper, we analyse school pupils’ performance on differentiating different kinds of cells and compare their performance with two neural networks (AlexNet and VGG16). It was observed that children tend to get very good performance in tumour cell annotation with the best F1 measure 0.81 which is a metrics taking both false positives and false negatives into account. Low accuracy was achieved with F1 score 0.75 on positive non-tumour cells and 0.59 on negative non-tumour cells. Superior performance on non-tumour cell detection was achieved by neural networks. VGG16 with training from scratch achieved an F1 score over 0.70 in all cell categories and 0.92 in tumour cell detection. We conclude that non-experts like school pupils have the potential to contribute to large-scale labelling for AI algorithm development if sufficient training activities are organised. We hope that competitions like this can promote public interest in pathology and encourage participation by more non-experts for annotation.

## Introduction

In March 2020, the WHO characterised coronavirus disease 2019 (COVID-19) as a pandemic due to a novel SARS-CoV-2 virus. On March 18, 2020, the United Nation (UN) Educational, Scientific and Cultural Organization estimated that 107 countries have implemented national COVID-19 related school closures, affecting roughly half the global student population. In the United States, all 50 states closed schools in March 2020 to reduce the spread of COVID-19. In the United Kingdom, schools were closed to all but key workers or vulnerable children in March 2020 and did not reopen until September 2020. Home schooling became the ‘’new normal’’ during this period and there were many challenges for schools, parents and children having never been in this position before. Rapidly, classes had to be moved online and remote learning undertaken in many countries across the world. The UK Government established the Oak National Academy with online classes for school children and created 10,000 lessons^1^. But many children still had spare time on their hands and parents struggled to find activities to entertain them during periods of lockdown and often were combining home-schooling with working from home. Many celebrities created online activities such as YouTube classes for Physical Exercise (PE). This triggered the PathLAKE consortium to consider whether it could contribute to these online activities whilst also promoting the collaborative work of pathologists and computer scientists in artificial intelligence (AI) by building a web-based competition for school age participants. The competition was promoted by the Royal College of Pathologists during the National Pathology Week 2020.

The PathLAKE consortium is one of the UK government’s five AI centres of excellence established in 2018 and funded by UK Research and Innovation (UKRI). PathLAKE is a digital pathology consortium and features university and NHS partners from Coventry, Warwick, Belfast, Nottingham and Oxford. Its aims include the creation of fully digital cellular pathology laboratories, the creation of an ethically approved data lake of anonymous scanned slide images and development of AI algorithms. The centres were created as part of the UK Government’s Industrial Life Sciences Strategy and pathology was described in this report as being ready for innovation. The use of digital pathology and AI to support the work of pathologists was highlighted^2^.

The interest in AI in cellular pathology has accelerated markedly in recent years. There are now many publications on algorithms that can support pathology workflow or predict the presence of gene mutations from morphological appearances^3^. In addition, commercially available algorithms exist which have regulatory clearance for diagnostic use, for example in prostate cancer detection^4,5^ although several challenges exist to their real-world uptake and use^6^. In order to test and train such algorithms, annotations for ground truth are required. Annotations can be derived in several ways, but one common method used is pixel level annotation whereby pathologists label histology images for particular objects of interest e.g. cancer cells. We therefore focused our online challenge on asking the competition participants to attempt to ‘’Beat the Pathologists’’ by annotating a series of images as an introduction to AI in pathology. These were images of breast cancer stained for Ki67 as a marker of proliferation and had already had annotations created by expert histopathologists. Participants’ annotation performance was then analysed and compared with neural networks.

The use of similar competitions, crowdsourcing and citizen science to promote pathology as a scientific discipline can be very helpful in addressing practical challenges related to recruitment into clinical pathology. The percentage of doctors choosing pathology as a career choice after qualification has fallen significantly in recent years and this trend is showing no signs of reversal^7–11^. The effect of this is indicated in various workforce census which show that only 3% of histopathology departments in the UK have enough staff to meet clinical demand^12^. In an effort to promote public interest in pathology, the Royal College of Pathologists (RCPath) organizes an annual international art competition called “Art of Pathology” which involves submissions of paintings, drawings, sculptures, digital art, collages or any other media on a specific theme related to pathology by participants of all ages including an under-11 category^13^.

Patient and public involvement (PPI) in research is vital to ensure that the work is acceptable to the public. Engagement activities can take many forms and previously described activities have involved participants in online interactions to develop or build algorithms, being involved in scientific experiments or classification of objects. Examples include a collaboration with the players of the Foldit computer game to generate cryo electron microscopy structures^14^, Etch a Cell whereby participants were asked to help with annotations of cellular structures within electron microscopy images^15^, a citizen science project to design de novo proteins^16^, a study to crowdsource image annotation for nucleus detection and segmentation^25^ and an astronomy project which asked the public to classify galaxies^17^. Over the past few years, hundreds of such citizen science projects have been conducted^18^. Systematic impact analyses show that such projects, especially science competitions, have had a significant positive influence on research advances and recruitment into science, technology, engineering and medicine (STEM) fields^19–23^. One of the most successful examples of such competitions is the annual International Genetically Engineered Machine (iGEM) competition which was launched in 2003 and has attracted international participation from thousands of participants and hundreds of teams and has led to the development of successful synthetic biology tools (https://igem.org/Main_Page)^24^.

In order to fill up pupils’ spare time and evaluate their ability to contribute to image annotations for AI development, in this study, we describe “Beat the Pathologists—Can you beat the experts?” competition which targeted school pupils from primary age to sixth form (4–18 years old). The different competition levels, from Mild to Supercharger, were designed to make the competition accessible to all whilst presenting a greater challenge to students as they moved to the higher levels. In this competition, participants were able to see several annotation examples done by pathologists, learn the introductory video which gives a guidance for differentiating particular objects of interest and try identifying (annotating) different types of cells. At the end of the competition, every participant received a Certificate of Participation and prizes were awarded to the top three scorers in each category (Primary (4–11 years old), Secondary (11–16 years old) and Sixth Form (16–18 years old)).

## Materials and methods

This study was conducted in accordance with approved guidelines. PathLAKE is a Research Ethics Committee (REC) approved research database, reference 19/SC/0363. In view of data governance restrictions, we collected only a username (not actual name) and category (primary (4–11 years old), secondary (11–16 years old), sixth form (16–18 years old). Informed consent from parents/guardians was obtained to acknowledgment that they accepted the terms and conditions. The full terms and conditions that were consented by parents/guardians is available in the Supplementary material.

The competition was launched twice since summer 2020. Based on feedback from the first edition, the competition setup and interface were improved to make it more user-friendly and understandable to school children. In this section, datasets, design of each edition (Pilot and Main) and additional source of annotation will be given in detail.

### Dataset

**Figure 1:**
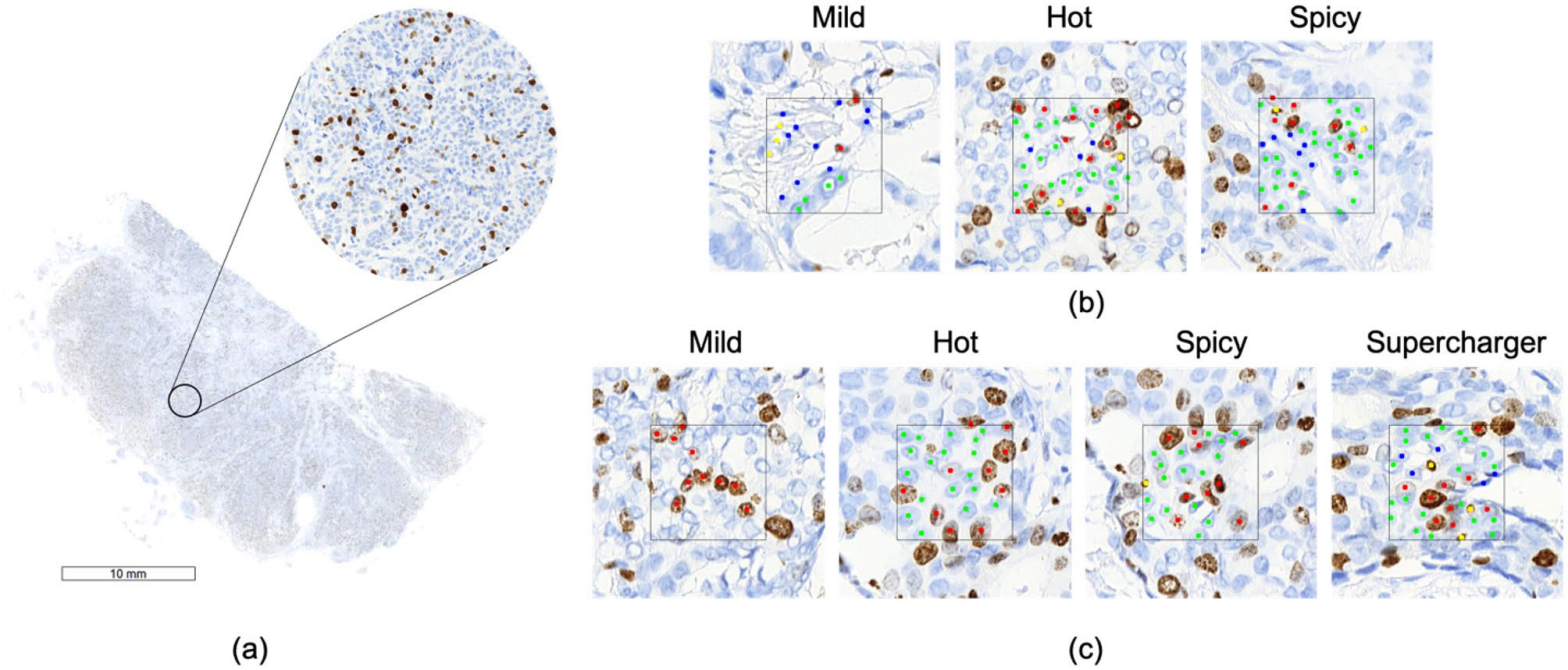
(**a**) One Ki67 stained WSI where one tumour region is zoomed in. (**b**) Example images of each level in Pilot competition. (**c**) Example images of each level in Main competition (Red: Positive Tumour or PT; green: Negative Tumour or NT; yellow: Positive Non-Tumour or PNT; blue: Negative Non-Tumour or NNT).

The images used in this study were of Ki67-stained whole slide images (WSI) (Fig. 1a) of ER-positive HER2-negative primary breast cancer from the PathLAKE project (Research Ethics Committee, reference number 19/SC/0363). Ki67 is a nuclear protein that is associated with cellular proliferation. From these WSIs, we extracted 512 × 512 pixel size images at 40X magnification from tumour regions. Images were annotated by expert breast pathologists [IMM, MT, MP, AK, AI] from Nottingham University Hospital and the annotations are taken as ground truth in this competition. 300 images were annotated by two pathologists each and good agreement was reached between pathologists. Four kinds of cells were annotated in this study: positive tumour cell (PT), negative tumour cell (NT), positive non-tumour cell (PNT) and negative non-tumour cell (NNT). In order to give the annotators enough context information, we limit the annotation only in the central 256 × 256 pixel square area.

### Pilot competition

A pilot phase of the competition was initiated in August 2020. Participants were school pupils and were asked to specify their participation category: primary (4–11 years old), secondary (11–16 years old), sixth form (16–18 years old). Generally, most of the participants did not have experience in cellular pathology. We provided instructions on the front-page of the competition to help the participants understand the task and grasp the cell identification knowledge in a short time. The instructions covered: what the four kinds of cells (PT, NT, PNT, NNT) look like, how to do the cell annotation using the web interface, the number of levels included in this competition. We also added an accompanying video from an expert pathologist (CV) from Oxford PathLaKE on the front-page to help the participants understand different cells and explain the relevance of Ki67 – with higher proliferation rates being associated with more aggressive behaviour^26^.

After having a general understanding of different categories of cells, participants were then guided to the practice part which had 50 images. Participants could go through all the images and in each image, identify four different kinds of cells. Annotations from the pathologists were available to the participants and regarded as ground truth in this competition. School pupils were able to evaluate their performance by comparing their annotations with the ones from pathologists. Each image could only be seen once.

The pilot competition was composed of three levels: Mild, Hot and Spicy. The levels were determined by the pathologist according to the difficulty and complexity of labelling different kinds of cells. Figure 1(b) gives several annotation examples of each competition level. Mild level contained images in which cells were fewer in number and with fewer combinations of cell types. Spicy level was the one with the highest difficulty. In addition, the number of images varied among different levels. Mild, Hot and Spicy level contained 20, 30 and 50 images respectively. Participants could not go directly to higher levels before successfully completing the previous ones. The condition of passing each level was that at least 50% of the cells in the current level had to be correctly annotated. Participants could enter each level as many times as they could to improve the annotation accuracy.

General feedback from participants on the Pilot edition was that it was quite difficult. Gradually increasing the complexity and number of cell categories with the increase of competition levels would help them to get the knowledge step by step.

### Main competition: “Beat the pathologists”

With the lessons that we learned from the Pilot phase, we improved the competition setup and re-launched the competition in October 2020 at the Oxford Science Festival. School pupils who registered covered primary (4-11 years old), secondary (11-16 years old) and sixth-form (16-18 years old) categories. They were trained through the Practice segment in preparation for the competition. The practice part was divided into two levels: Mild and Hot. Each level contained 5 images. In Mild level, pupils were guided to only annotate one kind of cell (PT). One more kind of cell which is NT was added to the Hot level. This modification was to help participants learn from basics and understand the appearance of different cells gradually. In addition, participants could access images in the practice part without time constraints.

There were four levels in the competition part: Mild, Hot, Spicy and Supercharger with 20, 40, 60 and 80 images respectively. As can be seen from Fig. 1(c), with each increase of level, one more cell type was added to increase the complexity of the annotations. Participants had to begin from Mild level and correctly annotate at least half of the cells so that they could continue to the next level. Table 1 gives a summary about the design differences between these two competition launches.

**Table 1:** Setup of the pilot and main competition.

	Launch time	No. of levels	No. of images	No. of cell categories
**Pilot**				
Practice	3rd August 2020–6th September 2020	1	50	4 in all images
Competition		3 (Mild, Hot, Spicy)	20, 30, 50 respectively	4 in all images
**Main**				
Practice	17th October 2020–16th November 2020	2	10	1 in 5 images and 2 in another 5 images
Competition		4 (Mild, Hot, Spicy, Supercharger)	20, 40, 60, 80 respectively	1, 2, 3, 4 respectively

### Sources of annotation

**Figure 2:**
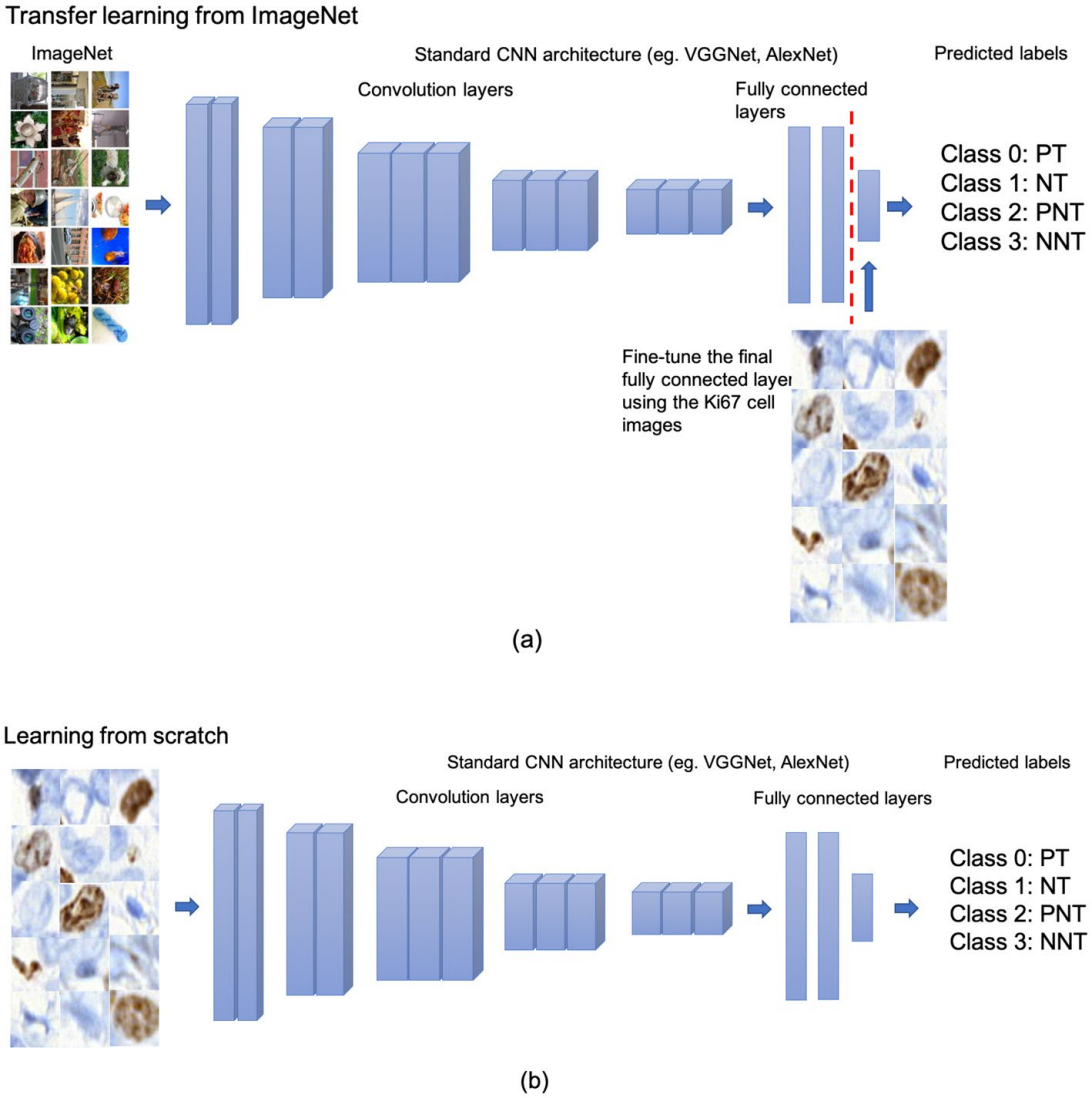
Illustrations of (**a**) transfer learning: a neural network is pretrained on ImageNet and subsequently trained on Ki67 cell images to perform 4 class classification (**b**) training from scratch: training the complete network from the first layer using the Ki67 cell images (PT: Positive Tumour; NT: Negative Tumour; PNT: Positive Non-Tumour; NNT: Negative Non-Tumour).

In addition to annotations from expert pathologists and those from school children, we obtained annotations from neural networks which were trained on the same number of images that were seen in the Practice session by the participants. Two popular neural networks (AlexNet^27^ and VGG16^28^) were evaluated in this comparison. School pupils only saw 10 cell images in the Practice part and 5 images in the introductory video. Therefore, we used the same 15 images to train the neural network and test the trained network on the images in the Supercharger level to understand how AI could perform when faced with the same training as the pupils. Two strategies were used here: transfer learning and training from scratch. In Fig. 2, the difference between these two strategies is plotted. Briefly, in transfer learning, the network is pre-trained on the ImageNet dataset^29^ which is a large visual database designed for use in visual object recognition software research. Some layers are frozen (left of the red dashed line) within the neural network and the final fully connected layer is fine-tuned using the aforementioned Practice Ki67 cell dataset. Instead of using the network weights pre-trained on ImageNet, learning from scratch involves training the whole network using the Practice Ki67 cell dataset only. Due to the limited number of inputs, we used data augmentation techniques including flip, rotation to increase the amount of data during the training.

### Ethical approval

PathLAKE is a Research Ethics Committee (REC) approved research database, reference 19/SC/0363. PathLAKE dataset will be made publicly available in the future. CV is part funded by the National Institute for Health Research (NIHR) Oxford Biomedical Research Centre (BRC). The views expressed are those of the author(s) and not necessarily those of the NHS, the NIHR or the Department of Health.

## Results

### Evaluation criteria

Cell annotation accuracy in the competition:

A cell was accepted as correctly annotated if the dotting coordinates were within a range of 14 pixels (i.e., within 3.5 µm—image scanned at X40 with a resolution of 0.25 µm/pixel) from the ground truth. The evaluation accuracy was calculated by dividing the total number of correct annotations by the total number of ground truth in each level. The accuracy ranged from 0 to 100 because the percentage was multiplied with 100 during the accuracy ranking.

$${\mathrm{Accuracy }}=\frac{{\mathrm{Number\, of\, correctly\, annotated\, cells}}}{{\mathrm{Number \,of \,all\, cells}}} \times 100$$

Further evaluation metrics:

In this paper, we further use F1 measure to evaluate the accuracy. Instead of only calculating the percentage of corrected annotated cells, F1 measure takes both false positives and false negatives into account. It is defined as the weighted average of precision and recall (F1 = 2 × TPR × PPV/(TPR + PPV)). TPR is recall or true positive rate (TPR = TP/(TP + FN)) and PPV is precision or positive predictive value (PPV = TP/(TP + FP)) where TP is the number of true positives, FP is the number of false positives and FN is the number of false negatives.

### Engagement and participation

As the pilot phase of the competition was advertised and launched during the summer time, many schools were not in an active connection with parents and pupils. A total of 28 pupils registered for the competition and finished the training task. Only 5 of them chose to participate in the competition. We believe that the low number who moved from practice to the competition was in part related to high degree of complexity in the practice and earlier competition levels. This was too much of a leap for pupils without any experience with cell annotation and pathology. Among the five participants, one pupil passed the Mild level and entered the Hot level. No participant progressed to the final Spicy level. Again, the difficulty level was felt to be a factor here. From the Pilot experience, we concluded that the participants needed a stepwise approach to understand the appearance of different categories of cells.

**Figure 3:**
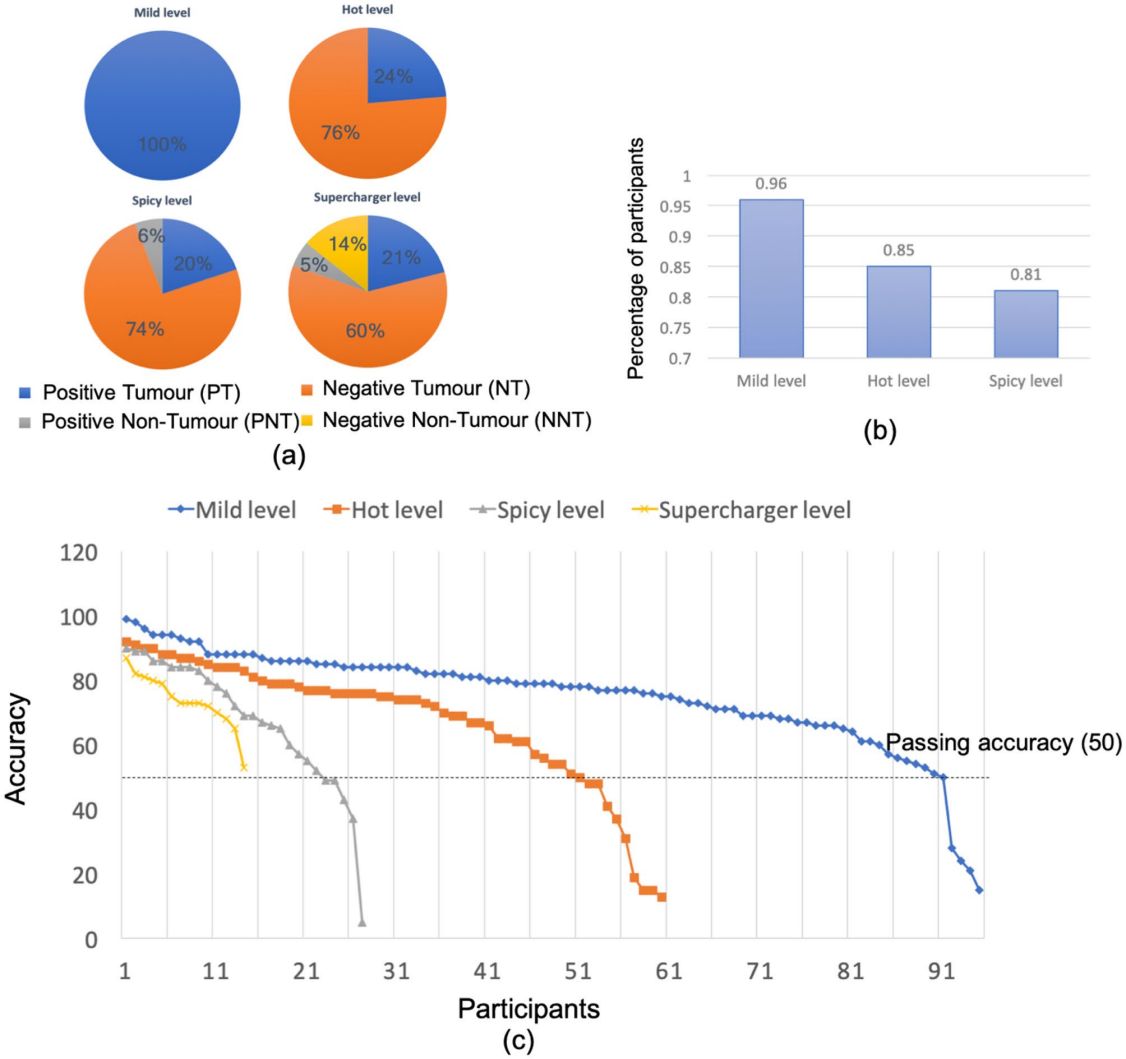
(**a**) Percentage of different kinds of cells in the four competition levels: Mild, Hot, Spicy and Supercharger. (**b**) Percentage of participants who pass the current level and join the next level. (**c**) Accuracy of all participants in the competition levels.

In the Main competition, we simplified the Practice part and the earlier competition levels. As a result, a total of 98 pupils registered for the competition and 95 of them (97%) continued to participate in the competition. Figure 3(a) gives the distribution of different kinds of cells in each competition level. One more category of cell was added to the annotations at each level, starting with the cells that were easier to recognise, i.e. positive tumour cells, moving to the most difficult—negative non-tumour cells. In the Supercharger level, which included four categories of cells, the majority (81%) of the cells were tumour cells, 60% were negative tumour cells and 21% positive tumour cells with the negative cells arguably being more difficult than the obvious brown coloured positive cells. We plotted the accuracy scores achieved by participants in each competition level in Fig. 3(b) and (c). The majority of participants (n = 91, 96%) obtained scores higher than the threshold 50 in the Mild level and 61 of them choose to continue the Hot level. 52 participants (85%) then passed the Hot level and 28 of them continued to join the Spicy level. Among the 28 participants who attempted the Spicy level, 22 of them (81%) successfully unlocked the final Supercharger level of the competition. This was a significant improvement from the pilot phase with the stepwise approach enabling participants to move more easily to the higher levels. It was good to see that with the increase in the level complexity, the percentage of participants who were able to unlock the higher level decreased. The top accuracy in each level also decreased from 99 to 87. This supported our design of the Main competition that the level sequence and cell annotation complexity were consistent. Relevant codes are available in https://github.com/TIA-Lab/pathcomp.

### Lessons learnt from the pilot phase of the competition

The successful launch of the main competition results from the lessons learnt from the pilot phase of the competition. They are concluded as follows:

**Gradual increase in the complexity of the tasks** Following the Pilot edition that run during summer 2020, we investigated why 82% participants had registered but not taken part in the competition and 80% who had started the competition could not get past the first level. We sought in depth input from 2 school age children (ages 10 and 12), asking them to participate in this competition. The feedback was that even after watching the introductory video and reading the instructions that they were still slightly unclear on what to do. The ‘’leap’’ straight into annotating 4 cell types in level 1 (Mild level) was too great and thus the suggestion from the two subjects was to introduce a new cell type in each level, starting with the simplest cell type (PT) and moving to the most difficult (NNT) as a step-wise way to gradually increase complexity. We also introduced such gradual learning in practice sessions.

**Launch timing** Launching the Pilot edition over the summer of 2020 with the aim of providing an activity over the summer turned out to be sub-optimal timing as many schools were not actively communicating with parents and children in order to promote the activity.

**Launch event** Launching the Main competition at the virtual Oxford Science Festival with a launch ceremony video helped promote the competition to a wider audience. In addition specific promotional materials aimed at schools were created to promote the competition.

### Comparison with pathologists

**Figure 4:**
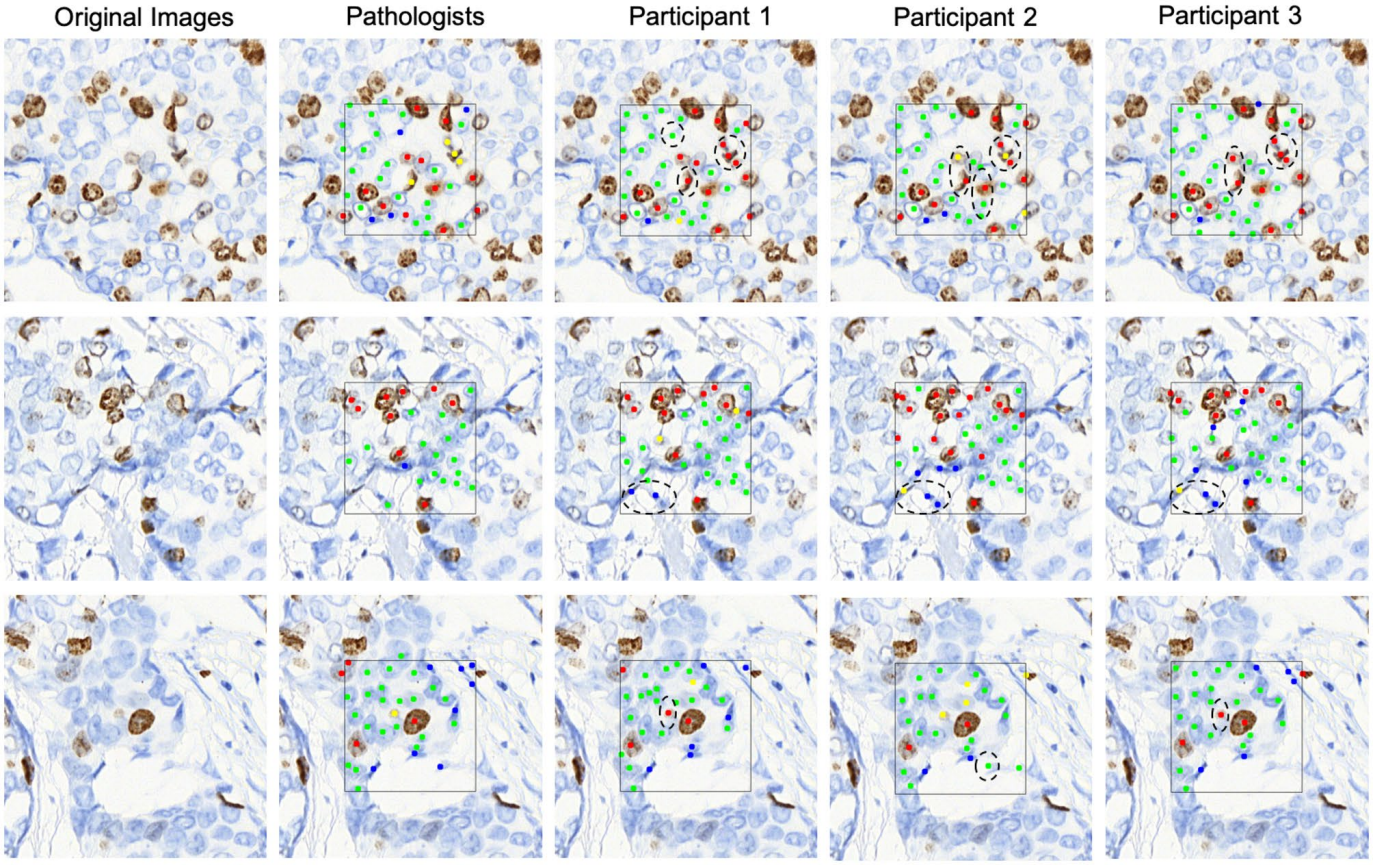
Examples of cell annotation results by the pathologist and three participants who achieved the top three accuracies in the Supercharger level. (Positive Tumour: red; Negative Tumour: green; Positive Non-Tumour: yellow; Negative Non-Tumour: blue). The annotations by the pathologists were used as ground truth.

In Fig. 4, we give some example images that were annotated by the pathologists and three participants who achieved the top three accuracies in the Supercharger level. Pathologist’s annotations were regarded as ground truth (GT). As observed, each participant could detect tumour cells with high accuracy. However, participants tended to confuse the positive non-tumour cells (yellow) with the positive tumour cells (red). Some of these mis-detections are denoted by black dashed circle and this would sometimes be a difficult and subjective distinction. Also, it may result from the lack of non-tumour cell training in the Practice part. In addition, some artefacts are mis-detected as cells.

The evaluation metrics on cell detection by participants who ranked top three in each level are shown in Table 2. Focusing on the F1 measure which incorporates both precision and recall, we observe that lower performance is achieved on non-tumour cell detection. As can be observed from the Supercharger level, F1 is 0.75 on PNT and 0.59 on NNT while 0.82 on PT and 0.80 on NT. It is mainly because the training on non-tumour cell identification is not sufficient and the difficulty level is high. We should add more training sessions about non-tumour cells, especially negative non-tumour cells in the Practice part in later launches of the competition.

**Table 2:** F1 score on cell detection by participants who ranked top three in each level.

Level	Cell	F1 score (1st)	F1 score (2nd)	F1 score (3rd)
Mild	PT	0.90	0.89	0.89
Hot	PT	0.88	0.88	0.87
	NT	0.84	0.84	0.82
Spicy	PT	0.84	0.84	0.81
	NT	0.82	0.80	0.80
	PNT	0.78	0.72	0.74
Supercharger	PT	0.82	0.78	0.74
	NT	0.80	0.80	0.81
	PNT	0.75	0.68	0.61
	NNT	0.59	0.60	0.61

**Figure 5:**
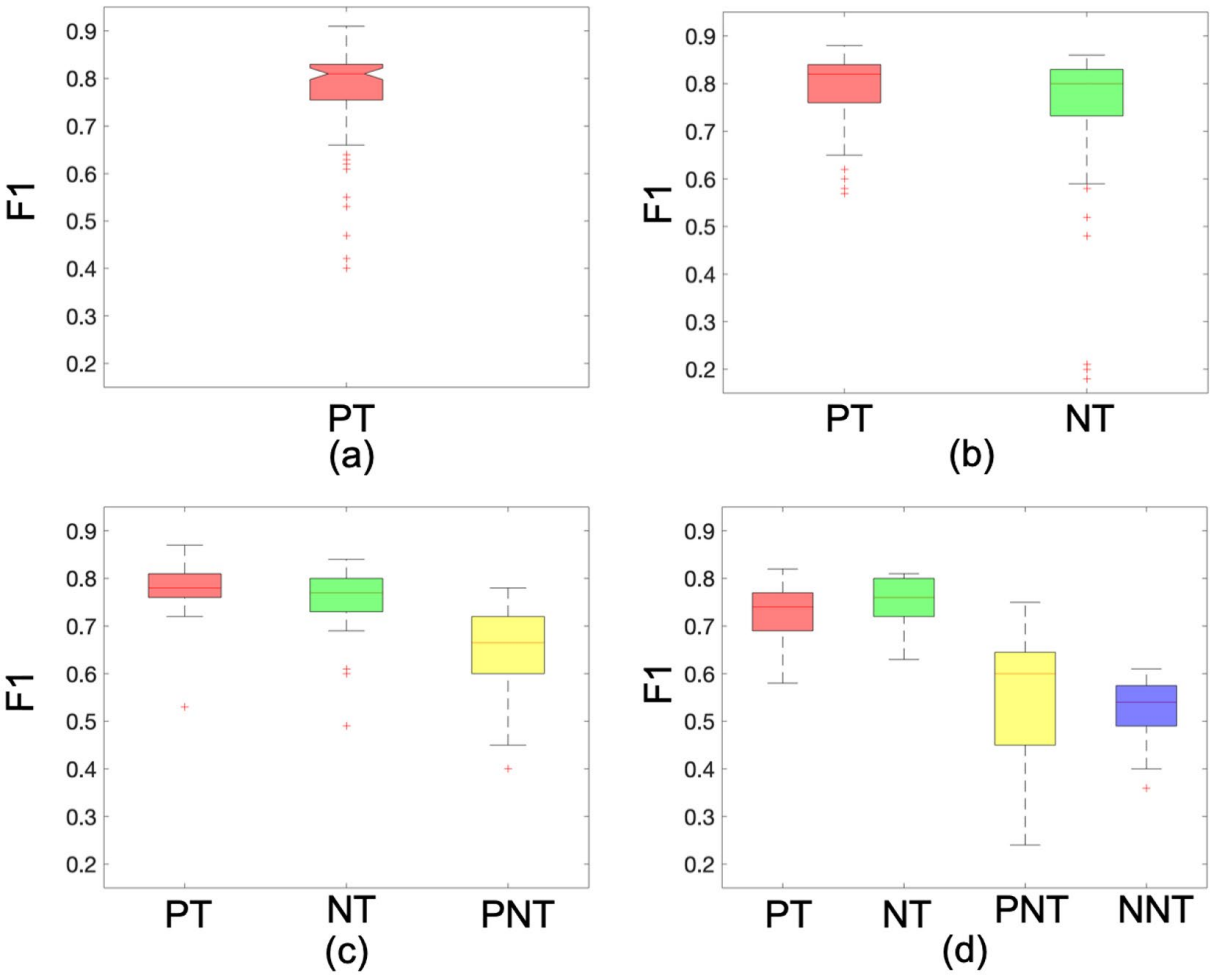
Distribution of F1 among different cell categories and competition levels. (**a**) Mild level; (**b**) Hot level; (**c**) Spicy level; (**d**) Supercharger level. (PT: Positive Tumour; NT: Negative Tumour; PNT: Positive Non-Tumour; NNT: Negative Non-Tumour).

In Fig. 5, we further evaluated the F1 distribution among all the participants in each competition level. It is clear to see that F1 measure gives a similar value (around 0.80) in both tumour cell identification. However, it appears more variance and lower mean value on non-tumour cell identification. In the Supercharger level, F1 on PNT is 0.60, ranging from 0.24 to 0.75 while F1 on NNT is 0.53 ranging from 0.36 to 0.61.

### Machine learning algorithm performance

**Figure 6:**
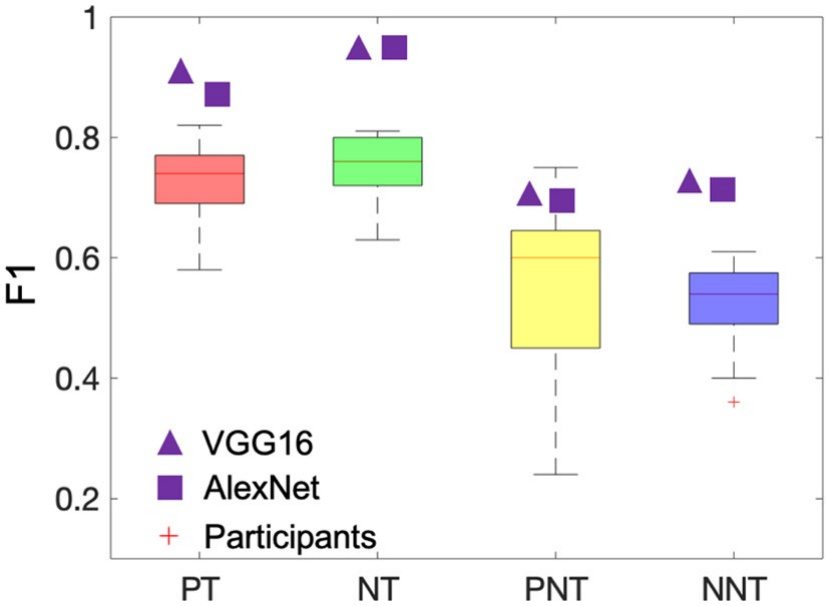
Comparison between the F1 achieved by participants and the one by neural networks which are trained from scratch in Supercharger level. Purple triangle represents the results using VGG16 while purple square represents the results by AlexNet.

We evaluated the competition performance of two popular neural networks after training on the same images as the pupils (AlexNet^27^ and VGG16^28^). Table 3 gives the cell detection performance using both neural networks and strategies. Similar results are observed between the two neural networks. It is interesting to see that compared with transfer learning, training from scratch gives a much higher F1, especially in non-tumour cell detection. VGG16 with training from scratch gives the best performance with F1 0.91 in PT, 0.93 in NT, 0.70 in PNT and 0.72 in NNT. We also plot the F1 achieved by neural networks with training from scratch and participants in Fig. 6. Same as we observed in Table 3, neural networks achieve higher F1 compared with the averaged F1 by participants in all the four cell categories. It should be noticed that in PT, NT and NNT, the neural networks which are trained from scratch exceed the best performance by the participants. These observations prove the learning superiority of neural networks.

**Table 3:** F1 score on cell detection using different neural networks and strategies.

Network	Pre-trained	F1 (PT)	F1 (NT)	F1 (PNT)	F1 (NNT)
AlexNet	Yes (ImageNet)	0.78	0.82	0.34	0.44
AlexNet	No	0.88	0.93	0.69	0.71
VGG16	Yes (ImageNet)	0.72	0.82	0.34	0.48
VGG16	No	**0.91**	**0.93**	**0.70**	**0.72**

Significant values are in bold.

## Discussion and conclusion

Due to the COVID-19 pandemic, nearly half the global student population have been affected by school closures. The closing of schools has shifted the site of learning to the home which brings many challenges to schools, parents and children. In order to fill up pupils’ spare time and evaluate their ability to contribute pathology image annotations for the development of AI models, we organised a competition named ‘Beat the Pathologists’ in which school pupils were encouraged to learn cell morphology and identify 4 different types of cells (positive tumour cell, negative tumour cell, positive non-tumour cell, negative non-tumour cell). Pupils were ranked based on their annotation accuracy. Every participant received a Certificate of Participation and prizes were awarded to the pupils who are ranked top three.

In this paper, taking pathologists’ inputs as ground truth, we analyse school pupils’ annotation results and compare their accuracy with two neural networks (AlexNet and VGG16). It is surprising to see that the pupil who ranked top in the Supercharger level achieved F1 as high as 0.81 in tumour cell annotation. However, lower performance is achieved on non-tumour cell detection with F1 0.75 on PNT and 0.59 on NNT. For non-tumour cell identification, more variance and lower mean values of F1 score were observed. A possible reason for this could be that training on non-tumour cell identification was not sufficient before the competition and the difficulty level of annotating non-tumour cells was relatively high. More training sessions for non-tumour cells, especially negative non-tumour cells could be added in the Practice part in later editions of the competition. Among the neural networks, VGG16 with training from scratch gave the best annotation performance with F1 0.91 in PT, 0.93 in NT, 0.70 in PNT and 0.72 in NNT. These values were higher than the averaged F1 achieved by participants in all cell types and exceed the best performance in PT, NT and PNT detection, which highlights the utility of neural networks to learn and undertake complex, repetitive tasks.

Given the pupils’ promising performance on differentiating different types of cells and limited training dataset available before the competition, it is reasonable to propose that school pupils have the potential to reach high cell annotation accuracy if sufficient training time and dataset are available. Annotations by pupils can be potentially useful in computational pathology where pathologists are often asked to annotate histology images for particular objects of interest e.g. cancer cells. Annotation is laborious and time consuming and the percentage of doctors choosing pathology as a career choice has fallen significantly in recent years. Similar competitions can be helpful in addressing such challenges and cultivating school pupils who can contribute to annotation for AI algorithm development.

In order to get school pupils sufficient training experience, it should be ensured that a variety of representative example images are present in the Practice part and introductory video providing a tutorial that covers detailed explanation of particular objects of interest in an easily understandable way. For the cells which are hard to identify, extra training images, practice and tutorials should be guaranteed. In addition, online activities like YouTube classes can be organised to introduce pupils daily pathology practice and experimental AI applications.

The competition was successful with 95 entrants in the Main edition and 15 participants succeeding in progressing to the final Supercharger level. We hope that such competitions can promote pupils’ interest in pathology and AI and pave the way for the promotion of collaborative work being undertaken by cellular pathologists with computer scientists to school pupils. The legacy will hopefully be a future generation of cellular pathologists inspired by interaction with ground-breaking work. To continue the story and build new knowledge for participants, a new competition will be launched focusing on a new annotation challenge to label glandular structures in digital pathology images, thus moving from cell to structure based annotations.

## Supplementary Information

Supplementary Information.
