# Supplementary material for: Lessons from a breast cell annotation competition series for school pupils
Source: Sci Rep. 2022 May 12;12:7792. doi: 10.1038/s41598-022-11782-9 (PMC9098471; doi:10.1038/s41598-022-11782-9)
Supplement: Supplementary file 1 — Supplementary Information. [file 41598_2022_11782_MOESM1_ESM.docx]

Terms and Conditions

1. PathLAKE’s “Beat the Pathologists” Competition (the “Competition”) is open to persons aged between 4 to 19 years of age on Monday 31st August 2020 (the closing date for entries) who are full time residents of the UK (including the Channel Islands and the Isle Of Man) except the children or close relatives of PathLAKE staff. PathLAKE reserves the right to request proof of age for entrants as well as parent’s or guardian's consent. Participants can enter as many times as they wish. No joint entries will be allowed.

2. Entry will be in 3 age categories based on school year during the academic year September 2019-September 2020:

Category 1: School years: Foundation to year 6 inclusive

Category 2: School years: Year 7-year 11 inclusive

Category 3: School years 12 & 13

3. To enter the Competition, entrants must annotate or label as many images from the “Beat the Pathologists” competition as possible

4. Entry is via the online entry form which can only be downloaded from the Pathcomp website.

5. The Competition entry must be by the child without assistance from any adults.

6. All entrants must register a user name (which should not be their actual name) and should ask their parent/guardian’s permission to enter the Competition. They and their parent/guardian must ensure that they have read the Terms and Conditions of the Competition. This must be confirmed by completing the tick box on the entry form. The email provided must enable the parent or guardian to be contactable to receive notification of their prize from 1st-8th September 2020 (see also Terms and Conditions 13).

7. All entrants must enter the Competition by entering online. Entries can only be accepted online; postal entries or entries sent over email will not be considered.

8. The website will close to entries at midday on 31st August 2020. Late, incomplete or illegible entries or entries will be disqualified. PathLAKE cannot take any responsibility for any technical failure or malfunction, including but not limited to any entry being delayed, lost or not properly registered or recorded.

9. PathLAKE may wish to produce some generic information about winners for publication purposes on their website and social media.

10. All entries will be judged in the first instance by calculation of scores. If any entries which qualify for a prize are of equal score, a random number generator will select the winning entries. An independent assessor will be used to oversee this process if required.

11. Prizes: The 3 highest scoring entries in each category will be awarded Amazon gift vouchers (amazon.co.uk) which will be sent to winners over email. The prizes in each category are as follows: First prize: £50, Second prize £30, Third prize £20. A maximum of 3 prizes per category will be awarded (see Terms and Conditions Rule 10, in the event of tie).

12. These dates are non-movable, but we reserve the right to change them due to editorial/production considerations.

13. Emails will be sent to the winners between the 1st and 8th September. If an entrant is unable to be contacted after reasonable attempts have been made to do so, PathLAKE reserves the right to offer their place to the next highest scoring entrant. Unsuccessful entrants will not be contacted. No feedback can be provided.

14. The prizes will be as stated. The prizes cannot be transferred and there is no cash alternative.

15. The PathLAKE decision as to the winner is final. No correspondence relating to the Competition will be entered into.

16. PathLAKE reserves the right to cancel the Competition or amend any of these rules at any stage if deemed necessary in its opinion, or if circumstances arise outside of its control. Any such changes will be duly communicated on the website. PathLAKE cannot be held responsible for any unforeseen events relating to the competition.

17. PathLAKE will only ever use personal details for the purposes of administering the Competition and in accordance with the PathLAKE privacy policy. PathLAKE will not publish them or provide them to anyone without permission. You can read more about the PathLAKE privacy policy on https://www.pathlake.org/privacy-notice/

18. The parent/guardian of an applicant is deemed to have accepted these Terms and Conditions when consenting to the application of the relevant entrant.

19. PathLAKE and/or any other organisation associated with the Competition accepts no liability for any damage, loss, liabilities (including direct, indirect, incidental, consequential or punitive damage), injury or disappointment incurred or suffered by you as a result of entering the Competition, not being able to enter into the Competition or accepting any of the prizes. Nothing shall exclude the liability of PathLAKE for: (i) fraudulent misrepresentation; (ii) for death or personal injury as a result of that party's negligence; or (iii) any other cause of action which cannot be limited or excluded under applicable law. In no event shall PathLAKE and/or any other organisation associated with the Competition’s total liability to you for all damages, losses, or causes of action exceed the price of the relevant prize.

20. If any of these clauses should be determined to be illegal, invalid or otherwise unenforceable then it shall be severed and deleted from these Terms and Conditions and the remaining clauses shall survive, remain in full force and effect.

21. These Terms and Conditions shall be governed by and construed in accordance with the laws of England and Wales. The courts of England and Wales shall have exclusive jurisdiction over any claim, dispute or matter arising under or in connection with these Terms and Conditions.
